# National trends in the prevalence of dementia in Medicare Advantage and Traditional Medicare

**DOI:** 10.1186/s12913-025-13404-2

**Published:** 2026-03-07

**Authors:** Mauricio Lopez-Mendez, David J. Meyers, Victoria Shier, Eric Jutkowitz

**Affiliations:** 1 Department of Health Services, Policy & Practice, Brown University School of Public Health, Providence, RI USA; 2 Schaeffer Center for Health Policy & Economics, University of Southern California, Los Angeles, CA USA; 3 Sol Price School of Public Policy, University of Southern California, Los Angeles, CA USA; 4 Center of Innovation in Long Term Services and Supports, Providence VA Medical Center, Providence, RI USA; 5 VA Evidence Synthesis Program Center, Providence VA Health Care System, Providence, RI USA

**Keywords:** Dementia prevalence, Medicare Advantage, Traditional Medicare, Health and Retirement Study, Plan switching

## Abstract

**Background:**

Little is known about how the prevalence of dementia in Medicare Advantage (MA) and traditional Medicare (TM) has changed over time. We examine prevalence of dementia in MA and TM overall and by race/ethnicity, the characteristics of these individuals within plans, as well as enrollment and switching rates between MA and TM between 2000 and 2014.

**Methods:**

Repeated cross-sectional study using eight waves from the Health and Retirement Study (HRS) linked to Medicare enrollment data. Sample includes HRS respondents ≥ 65 years of age (*n* = 18,381) linked to Medicare enrollment data. Measurements used include predicted cognitive function (Langa-Weir classification: dementia, cognitive impairment not dementia, and normal), three race/ethnicity categories (White, Black, and Hispanic), demographic and clinical characteristics from HRS, and Medicare enrollment (MA or TM) per year.

**Results:**

Among TM enrollees, prevalence of dementia was lower by 4% points in 2014 (9.0%, 95%CI: 7.8%, 9.3%) compared to 2000 (13.0%, 95%CI: 12%, 14%). The prevalence of dementia in MA was higher by 2% points in 2014 (10.0%, 95%CI: 8.5%, 11%) compared to 2000 (8.0%, 95% CI: 7.2%, 9.7%). Prevalence of dementia in MA remained stable for Whites non-Hispanic, was 2% points higher for Blacks non-Hispanic, and 5% points higher for Hispanics in 2014 compared to 2000. MA compared to TM beneficiaries with dementia in 2014 were younger (mean [SE] 81.6 [0.5] vs. 83.5 [0.4]), had fewer activity of daily living limitations (1.9 [0.1] vs. 2.4 [0.1]), instrumental activities of daily living limitations (2.3 [0.1] vs. 2.8 [0.1]), and of chronic conditions (3.2 [0.1] vs. 3.5 [0.1]). By 1-year (2012–2013), 6.3% of MA beneficiaries with dementia switched to TM and 4.3% of TM beneficiaries with dementia switched to MA.

**Conclusions:**

Between 2000 and 2014, dementia prevalence was lower in TM compared to MA. Evidence suggests that MA beneficiaries with dementia are younger and have fewer functional limitations than their dementia TM counterparts.

**Supplementary Information:**

The online version contains supplementary material available at 10.1186/s12913-025-13404-2.

## Background

By 2060, 13.8 million people in the United States (US) are expected to be living with dementia, more than double the number in 2022 [1]. The majority of people living with dementia are enrolled in Medicare, which is a federal health insurance program for people ≥ 65 years of age.

Most of the research on the health care utilization of people with dementia in Medicare come from Traditional Medicare (TM) data. Yet, as of May 2023, over 50.0% of Medicare beneficiaries are enrolled in Medicare Advantage (MA) [2], which is the privately operated segment of Medicare. MA encounter data has only become available in recent years, limiting our understanding of the characteristics of beneficiaries with dementia enrolled in MA [3, 4].

There are important differences between the MA and TM programs that may affect the outcomes of beneficiaries with dementia as well as their plan choices. Unlike TM, MA plans can offer supplemental services (e.g., caregiver support or case management) that may benefit beneficiaries with dementia, and MA plans may be better at coordinating care. Simultaneously, MA may have higher cost sharing for certain services, restricted provider networks, and prior authorization policies that can create barriers to care for people with dementia [5]. From a policy perspective, there is also concern that MA organizations may engage in favorable selection (i.e., they enroll healthier beneficiaries whose care costs less than average) [6]. Given the growing popularity of MA and the expected increase in the number of people living with dementia, it is critical to understand the national prevalence of dementia in MA and TM, the characteristics of beneficiaries in these programs, and switching patterns between programs.

Existing studies have compared the prevalence and characteristics of people with dementia in MA and TM. For example, one study using 2014 data from three large MA plans found the diagnosed prevalence of dementia was 5.6%, which was lower than the prevalence of dementia in TM at the time [7]. The same study found that one year after enrolling in an MA plan nearly 32.0% people with dementia had disenrolled from the plan. A second study using Medicare enrollment data combined with health assessments also found substantially higher disenrollment among MA beneficiaries with dementia, and a third study found significantly higher disenrollment among Black and Hispanic beneficiaries with dementia [8, 9]. Disenrollment may be an important indicator of poor experience of care in each plan. Other studies have found that MA beneficiaries with dementia report worse care experiences than beneficiaries with other chronic conditions and that beneficiaries with dementia may be underrepresented in MA quality measurements [10]. Finally, other work has found that beneficiaries with dementia receive less costly care in MA compared to TM at the end of life [5, 11].

However, most of the above studies are limited to selected samples and/or restricted to characteristics available in Medicare enrollment records. In this study, we linked the Health and Retirement Study (HRS) to Medicare data to provide national estimates of dementia prevalence in TM and MA over time, compare the clinical and demographic characteristics of these beneficiaries, as well as enrollment and plan switching rates into TM and MA. We use HRS cognitive function measures to identify people with dementia in TM and MA. By using self-reported survey and performance-based measures from HRS to identify people living with dementia, this approach overcomes the limitations of claims-based approaches, which may underestimate the true prevalence [12].

## Methods

### Sample and data

First, we used data from eight waves (2000 to 2014) of the HRS, a nationally representative survey of United States adults over age 50. Individuals (or a proxy) participate in the survey every two years, which collects data on health and well-being, cognitive function, employment, and wealth. HRS respondents are followed until death and new cohorts are added to preserve the national representativeness of the survey. Second, we used the Medicare Claims and Summary cross reference [13] file to link HRS respondents to their Medicare claims. Using the linked HRS and Medicare data, we identified HRS respondents aged ≥ 65 years who were enrolled in MA or TM.

### Cognitive function measurement

We used the Langa-Weir classification algorithm to determine whether HRS respondents were predicted to have dementia, cognitive impairment not dementia (CIND), or normal cognitive function. The Langa-Weir algorithm generates a total cognitive score (27-point scale) based on HRS respondent answers to the immediate/delayed recall tests, serial 7’s test, and backward count for 20 tests [14]. For respondents with a proxy, memory, physical function, and the proxy’s assessment of memory are used to create a total cognitive score on an 11-point scale. In a validation study using a 3–4 h neuropsychological and clinical assessment combined with expert clinician adjudication as the gold standard, the Langa-Weir algorithm correctly classified 78.0% of all respondents, 76.0% for self-respondents, and 84.0% of those represented by a proxy [15]. 

### Medicare enrollment and dual eligibility

Using the Master Beneficiary Summary File, we identified the Medicare enrollment status of HRS respondents per calendar year. We classified a respondent as enrolled in MA if they had at least one month of MA enrollment and were never enrolled in TM during the year. A beneficiary with at least one month of TM enrollment and no MA enrollment in a year was classified as enrolled in TM. In sensitivity analyses, we ran analysis using a criterion of enrollment that only includes beneficiaries with 12 months of continuous enrollment in TM or MA during a calendar year, excluding those with partial enrollment (e.g., excluding those who die during a given year). We identified dually eligible beneficiaries (i.e., Medicare enrollee who is also eligible for Medicaid benefits) in three periods: 2000–2001, 2005–2006, and 2012–2013. We classified dually eligible Medicare beneficiaries by determining whether they were eligible to receive Medicaid benefits for at least one month during the first year of each two-year period, i.e., 2000, 2005, 2012 [16]. 

### Demographic and clinical characteristics

We obtained the following demographic characteristics from the HRS: age, sex, and race/ethnicity. We also obtained from the HRS the number of activities of daily living (ADL) limitations, number of instrumental activities of daily living (IADL) limitations, number of chronic health conditions, indicators of individual health conditions for diabetes, heart disease, hypertension, cancer, lung disease, arthritis, and psychiatric problems, and indicators for nursing home residence at time of HRS interview, and proxy respondents.

### Analysis

We descriptively compared the demographic and clinical characteristics of people with dementia in 2000 and 2014 and by Medicare enrollment status (estimates for in-between years are presented in the Appendix). To investigate favorable selection of healthier beneficiaries with dementia into MA plans, we compared the clinical characteristics of people with dementia between TM and MA beneficiaries stratified in three age groups (65–74; 75–84; and > = 85 years).

We computed annual national dementia prevalence estimates for the eight years of HRS waves between 2000 and 2014. We highlight results for years 2000 and 2014, estimates for in-between years are presented in the Appendix. For a given year, the numerator of the prevalence estimator was the total number of HRS respondents predicted to have dementia. The denominator of the prevalence estimator was the total number of HRS respondents in the analytic cohort. We cross-tabulated prevalence estimates by Medicare enrollment status, cognitive function, and race/ethnicity (White non-Hispanic, Black non-Hispanic, and Hispanic). For prevalence and enrollment analyses we used the HRS sampling weights to adjust for the sampling design of HRS and nonresponse.

Finally, we calculated rates of switching between TM and MA by cognitive function in the two-year period between 2012 and 2013 and stratified by dual eligibility. In the first year of the two-year period, we identify beneficiaries enrolled in MA or TM, their cognitive function status, and dual eligibility. To estimate switching rates, we track the changes in enrollment status and survival over the second year among beneficiaries identified in the first year of the period. To isolate the effect of different mortality rates between dementia and non-dementia groups in the interpretation of the switching rates, we compute the percentage of those who switched conditional on having survived into 2013 and compare them to estimates without conditioning on survival into 2013.

## Results

### Sample characteristics

Of the 20,175 unique respondents ≥ 65 years of age in HRS between 2000 and 2014, 18,381 (91.1%) respondents met our study inclusion and could be linked to Medicare enrollment data. Table 1 shows the characteristics of TM and MA beneficiaries with dementia in 2000 and 2014. Estimates for in-between years show similar trends in beneficiary characteristics (see Supplemental Table 1), and below we highlight data from 2000 to 2014.

**Table 1:** Characteristics of Medicare beneficiaries living with dementia (2000 vs. 2014)^*^

	Traditional Medicare		Medicare Advantage	
Characteristics	2000	2014	2000	2014
N	1,081	639	193	377
Age, yrs				
65–74	243 (23.3%)	98 (17.6%)	47 (25.8%)	64 (19.2%)
75–84	425 (42.0%)	251 (34.1%)	90 (46.2%)	182 (44.9%)
>= 85	413 (34.7%)	290 (48.3%)	56 (28.0%)	131 (35.9%)
Mean(SE)	81.0 (0.3)	83.5 (0.4)	79.8 (0.7)	81.6 (0.5)
Female	679 (64.0%)	408 (63.4%)	119 (62.2%)	232 (58.2%)
Race/Ethnicity^†^	1,081	638	193	377
White non-Hispanic	655 (70.5%)	409 (70.5%)	128 (76.3%)	202 (60.3%)
Black non-Hispanic	281 (18.7%)	130 (14.4%)	41 (15.1%)	97 (20.0%)
Hispanic	109 (7.4%)	82 (11.7%)	22 (8.2%)	74 (18.2%)
Net worth ^‡^				
Mean(SE)	143,803 (13,030)	251,441(24,125)	132,855(16,845)	344,060 (108,561)
Median(25th pct.,75th pct.)	36,500 (531;136,000)	75,000 (1,500; 246,500)	74,800 (3,000;185,129)	52,000 (1,000;211,000)
ADL Limitations ^§^				
0	403 (35.9%)	224 (37.4%)	89 (43.6%)	171 (46.4%)
1–3	336 (31.1%)	179 (37.4%)	59 (32.2%)	105 (27.5%)
4–6	342 (33.0%)	236 (35.9%)	45 (24.2%)	101 (26.0%)
Mean(SE)	2.3 (0.1)	2.4 (0.1)	1.9 (0.2)	1.9 (0.1)
IADL Limitations ^¶^	1,028	607	181	355
0	275 (24.8%)	152 (24.9%)	65 (32.1%)	122 (33.3%)
1–3	311 (29.8%)	140 (25.5%)	50 (28.0%)	97 (28.6%)
4–5	442 (45.4%)	315 (49.7%)	66 (39.9%)	136 (38.1%)
Mean(SE)	2.7 (0.1)	2.8 (0.1)	2.3 (0.2)	2.3 (0.1)
Cumulative Chronic Health Conditions (ever diagnosed)				
0	57 (5.7%)	5 (0.7%)	26 (14.0%)	10 (3.4%)
1–3	698 (65.2%)	286 (49.2%)	120 (61.9%)	205 (56.1%)
4–5	174 (16.8%)	157 (25.6%)	20 (9.7%)	80 (21.4%)
6–8	132 (12.3%)	151 (24.5%)	22 (14.4%)	66 (19.2%)
Mean(SE)	2.7 (0.1)	3.5 (0.1)	2.5 (0.1)	3.2 (0.1)
Individual Chronic Health Conditions (ever diagnosed)				
Stroke	308 (29.8%)	212 (30.9%)	52 (29.8%)	100 (27.5%)
Diabetes	222 (19.1%)	226 (33.7%)	46 (23.9%)	145 (38.4%)
Heart Disease	444 (41.4%)	299 (46.5%)	71 (39.7%)	137 (37.8%)
Hypertension	643 (58.4%)	511 (78.9%)	100 (49.9%)	305 (80.0%)
Cancer	137 (13.3%)	164 (25.6%)	33 (16.9%)	82 (20.5%)
Lung Disease	134 (13.3%)	102 (17.7%)	16 (9.2%)	63 (16.9%)
Psychiatric Problems	279 (26.7%)	232 (36.7%)	36 (20.8%)	111 (29.6%)
Arthritis	754 (67.9%)	515 (80.6%)	111 (58.2%)	280 (73.6%)
Live in Nursing Home During HRS Interview				
Yes	282 (29.9%)	195 (26.4%)	33 (20.9%)	67 (14.2%)
Respondent Type				
Proxy	557 (54.1%)	310 (46.0%)	77 (43.1%)	135 (33.8%)
Dually Eligible	NA	408 (34.4%)	NA	130 (28.8%)

^*^Note: In-between years (2006 and 2008) are presented in the Appendix (Supplemental Table 1). Percentages in parenthesis are weighted proportions derived using the survey sampling weights of the HRS. Enrollment Criteria: (a) Medicare Advantage: months enrolled in TM = 0 and months enrolled in MA > 0; (b) Traditional Medicare: months enrolled in TM > 0 and months enrolled in MA = 0. Abbreviations: ADL, activities of daily living; IADL, instrumental activities of daily living

^†^ Due to small number of respondents (< 25) we exclude the count of the category “other” for Race/ethnicity

^‡^ Includes all wealth components minus all debt components assessed in HRS

^§^ ADL Limitations: eating, transferring, toileting, dressing, bathing, and walking across a room

^¶^ IADL Limitations: preparing meals, grocery shopping, making phone calls, talking medications, managing money

In 2014 and compared to TM beneficiaries with dementia those enrolled in MA were younger (mean [SE] 81.6 [0.5] vs. 83.5 [0.4]), less likely to be female (58.2% vs. 63.4%), more likely to be Black non-Hispanic (20.0% vs. 14.4%) and Hispanic (18.2% vs. 11.7%). MA beneficiaries with dementia also had fewer ADL limitations (1.9 [0.1] vs. 2.4 [0.1]), IADL limitations (2.3 [0.1] vs. 2.8 [0.1]), and chronic conditions (3.2 [0.1] vs. 3.5 [0.1]) compared to those enrolled in TM. Finally, MA beneficiaries with dementia were less likely to be living in a nursing home at the time of their HRS interview (14.2% vs. 26.4%) or have a proxy respondent (33.8% vs. 46.0%) compared to TM beneficiaries with dementia.

The differences between MA and TM beneficiaries with dementia were not sensitive to controlling for age. Among Medicare beneficiaries living with dementia and age 65 to 74, the proportion of TM enrollees with 4–6 ADL limitations (worst category) was 21.7%(95%CI: 16.3%, 28.4%) in 2000 and 23.6%(95%CI: 15.1%, 35.0%) in 2014, while the proportion of MA enrollees with 4–6 ADL limitations decreased in 2000 was 24.4%(95%CI: 12.9%, 41.1%) and 19.1%(95%CI: 10.3%, 32.7%) in 2014. (Supplemental Table 2). Simultaneously, the proportion of TM enrollees with 4–5 IADL limitations (worst category) in 2000 was 30.0%(95%CI: 23.6%, 37.3%) and 26.3%(95%CI: 17.5%, 37.6%) in 2014, while the proportion of MA enrollees with 4–5 IADL limitations in 2000 was 31.9%(95%CI: 18.1%, 49.8%) and 18.7%(95%CI: 9.9%, 32.6%) in 2014. Finally, among Medicare beneficiaries in the same age group, the proportion of TM enrollees with 6–8 chronic health conditions (worst category) was 13.8%(95%CI: 9.5%, 19.6%) in 2000 and 27.2%(95%CI: 17.6%, 39.6%) in 2014, while the proportion of MA enrollees with 6–8 chronic health conditions in 2000 was 19.4%(95%CI: 9.3%, 36.1%) and 17.0%(95%CI: 8.8%, 30.2%) in 2014 (see Supplemental Table 2 for the same breakdown for the subsequent age categories).

### Annual prevalence of dementia within TM and MA

**Fig. 1:**
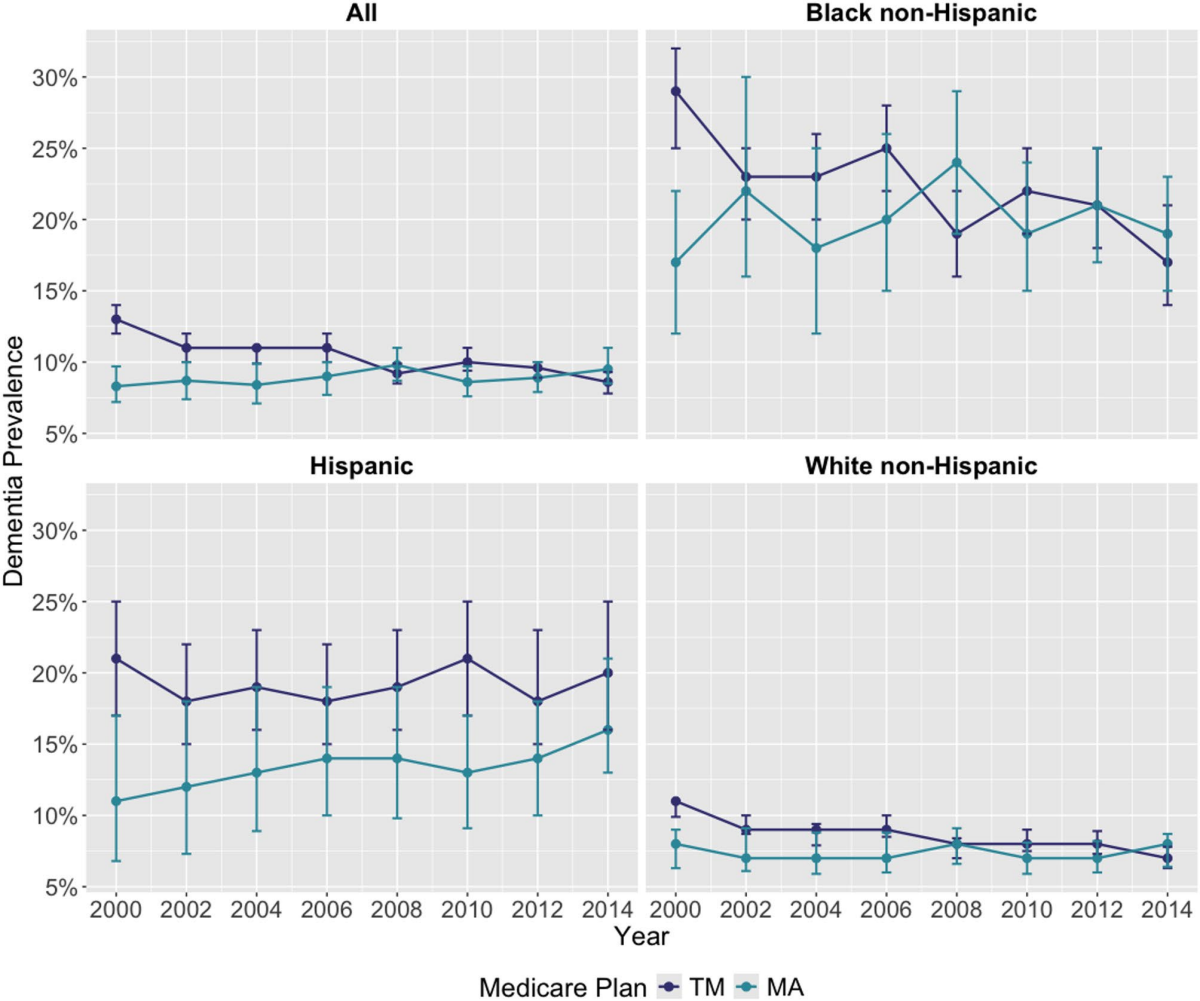
Dementia Prevalence between 2000–2014 (95%CI), by Medicare Enrollment and Race/Ethnicity. Annual prevalence is computed as the total number of HRS respondents predicted to have dementia in a year (numerator) divided by the total number of HRS respondents in the same year (denominator). Abbreviations: MA, Medicare Advantage; TM, Traditional Medicare. Enrollment Criteria: (a) Medicare Advantage: months enrolled in TM = 0 and months enrolled in MA > 0; (b) Traditional Medicare: months enrolled in TM > 0 and months enrolled in MA = 0. * Percentages are weighted proportions derived using the survey sampling weights of the HRS. Due to small number of respondents (< 25) we exclude the category “other” for race/ethnicity

The overall prevalence of dementia among Medicare beneficiaries ≥ 65 years of age was 12.0% (95%CI: 11.3%, 12.7%) in 2000 and 9.0% (95%CI: 8.4%, 9.6%) in 2014. Among TM enrollees, the overall prevalence of dementia was lower by 4% points from 2000 (13.0% 95%CI: 12.0%, 14.0%) to 2014 (9.0%, 95%CI: 7.8%, 9.3%). The prevalence of dementia in TM was also lower for White non-Hispanic, Black non-Hispanic, and Hispanic beneficiaries (Fig. 1).

Among beneficiaries in MA, the prevalence of dementia was higher by 2% points in 2014 (10.0%, 95%CI: 8.5%, 11.0%; Fig. 1) compared to 2000(8.0%, 95%CI: 7.2%, 9.7%). Among White non-Hispanic MA beneficiaries, the prevalence of dementia remained stable at 8% but was higher among Black non-Hispanic and Hispanic MA beneficiaries over the same period. Specifically, for Black non-Hispanic MA beneficiaries the prevalence of dementia was higher by 2% points in 2014 (19.0%, 95%CI: 15.0%, 23.0%) compared to 2000 (17.0%, 95%CI: 12.0%, 22.0%). Among Hispanics, the prevalence of dementia in MA was higher by 5% points in 2014 (16.0%, 95%CI: 13.0%, 21.0%) compared to 2000 (11.0%, 95%CI: 7.0%, 17.0%).

### Medicare Advantage enrollment by cognitive function status

**Fig. 2:**
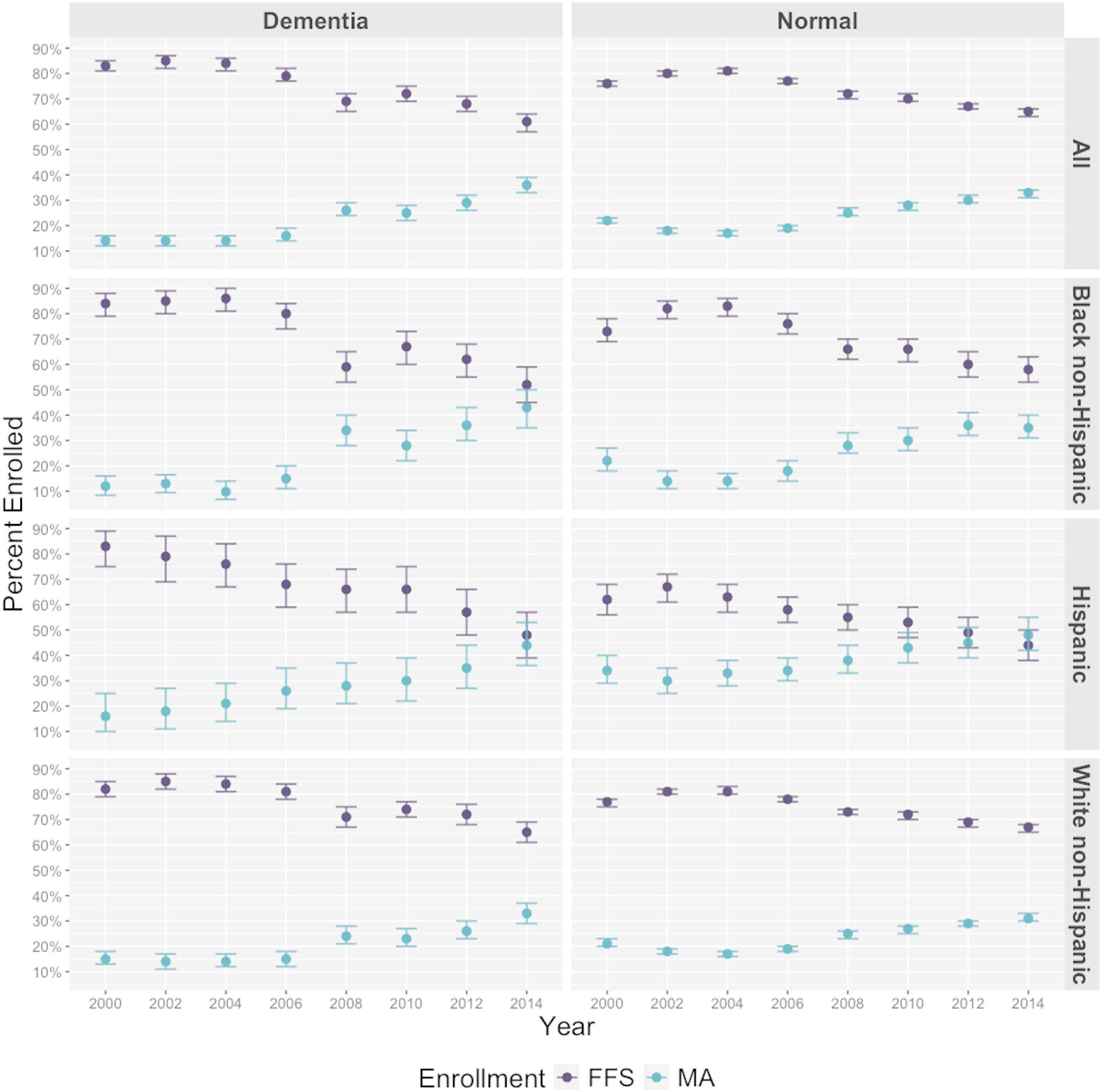
MA Enrollment between 2000–2014, by Cognitive Function and Race/Ethnicity. Annual enrollment is computed as the number of Medicare beneficiaries predicted to have dementia in the HRS enrolled to TM or MA in a given year (numerator), divided by the total number Medicare beneficiaries in that same year. Abbreviations: MA, Medicare Advantage; TM, Traditional Medicare. Enrollment Criteria: (a) Medicare Advantage: months enrolled in TM = 0 and months enrolled in MA > 0; (b) Traditional Medicare: months enrolled in TM > 0 and months enrolled in MA = 0. We exclude “Other” enrollment, which refers to cases whose enrollment was not classified as TM or MA based on the criteria for enrollment (< 3% of observations). * Percentages are weighted proportions computed using the survey sampling weights of the HRS. ^†^ Due to small number of respondents (< 25) we exclude the count of the category “other” for race/ethnicity

Among all Medicare beneficiaries with dementia, the proportion enrolled in MA was higher by 22% points in 2014 (36.0%, 95%CI: 33.0%, 39.0%; Fig. 2) compared to 2000 (14.0%, 95%CI: 12.0%, 16.0%). The difference in MA enrollment among people with dementia over the 14-year period was greater for Blacks non-Hispanic (+ 31.0%) and Hispanics (+ 38.0%) compared to Whites non-Hispanic (+ 18.0%).

Among beneficiaries with normal cognitive function, the proportion enrolled in MA was higher by 11% points in 2014 (33.0%, 95% CI: 31.0%, 34.0%) relative to 2000 (22.0%, 95%CI: 21.0%, 23.0%). The subpopulation with normal cognitive function with the largest difference in MA enrollment between 2000 and 2014 were Hispanics (+ 14.0%), followed by Blacks non-Hispanic (+ 13.0%) and Whites non-Hispanic (+ 10.0%). Beneficiaries with CIND showed similar trends to those with normal cognitive function (Appendix Supplemental Fig. 1). Results were consistent in the sensitivity analysis that only included beneficiaries with 12 months of continuous enrollment (TM or MA) during a calendar year (Appendix Supplemental Fig. 2).

### Medicare plan switching by cognitive function status and dual eligibility

Table 2 shows mortality and switching rates from 2012 to 2013 between TM and MA, stratified by cognitive function and dual eligibility. We observed that 6.3% of beneficiaries with dementia who enrolled in MA in 2012 and were alive in 2013 switched to TM in 2013. In contrast, 2.6% of beneficiaries with normal cognitive function who enrolled in MA in 2012 and were alive in 2013 switched to TM in 2013. Importantly, we observed no difference in switching to MA in 2013 between beneficiaries with dementia (4.3%) and those with normal cognitive function (4.3%), among those enrolled in TM in 2012 and alive in 2013.

**Table 2:** Plan switching 2012–2013^*^, by cognitive function (Dementia, Normal) and dual eligibility

		Mortality^+^		Switching Conditional on 1-year Survival^++^		
		Total Alive 2012	Died 2013	Total Alive 2012–2013	Enrolled in MA 2013	Enrolled in TM 2013
	Cognitive Function	n	%	n	%	%
Medicare Only and Dually Eligible Beneficiaries						
Enrolled in MA 2012	Normal	2237	3.5	2158	97.4	2.6
	Dementia	330	21.5	270	93.7	6.3
Enrolled in TM 2012	Normal	4778	3.7	4602	4.3	95.7
	Dementia	829	23.5	634	4.3	95.7
Medicare Only Beneficiaries						
Enrolled in MA 2012	Normal	2045	3.3	1977	97.5	2.5
	Dementia	250	22.0	195	92.8	7.2
Enrolled in TM 2012	Normal	4413	3.6	4254	3.9	96.1
	Dementia	533	24.8	401	3.5	96.5
Dually Eligible Beneficiaries						
Enrolled in MA 2012	Normal	192	5.7	181	96.1	3.9
	Dementia	91	17.6	75	96.0	4.0
Enrolled in TM 2012	Normal	365	4.7	348	8.9	91.1
	Dementia	296	21.3	233	5.6	94.4

Dually eligible Medicare beneficiaries were eligible to receive Medicaid benefits for at least one month during 2012. *MA* Medicare Advantage, *TM* Traditional Medicare, *CIND* cognitive impairment not dementia, dementia, Alzheimer’s disease and Alzheimer’s related Dementia. Enrollment Criteria: (a) Medicare Advantage: months enrolled in TM = 0 and months enrolled in MA > 0; (b) Traditional Medicare: months enrolled in TM > 0 and months enrolled in MA = 0. We exclude “Other” enrollment, which refers to cases whose enrollment was not classified as TM or MA based on the criteria for enrollment (< 3% of observations). ^+^Mortality is computed as the proportion of those who died in 2013. ^++^ Switching (conditional on 1-year survival) is computed by dividing the number of beneficiaries who switched plans by the total number of beneficiaries who survived into 2013. * Results for CIND cognitive function groups are available in the Appendix, Supplemental Table 4

Medicare-only beneficiaries (excluding those dually eligible) with dementia and normal cognitive function had similar switching patterns as described above. Switching patterns were different among the dually eligible population. Among those enrolled in MA in 2012 and alive in 2013, we observed a small difference in switching to TM in 2013 between beneficiaries with dementia (3.9%) and those with normal cognitive function (4.0%). Notably, among those enrolled in TM in 2012 and alive in 2013, we observed that 8.9% of beneficiaries with normal cognitive function switched to MA in 2013, while 5.6% of beneficiaries with dementia switched to MA in 2013.

Finally, among Medicare-only beneficiaries in 2012, the percent who died in 2013 was consistently higher among beneficiaries with or without dementia enrolled in TM than their counterparts in MA. Among dually eligible beneficiaries, only those with dementia in TM had a higher mortality than those in MA.

## Discussion

In this study we compared the annual prevalence of dementia between TM and MA populations over time, their clinical characteristics, and the annual enrollment and plan-switching patterns into TM or MA by cognitive function status. Similar to prior studies, we found the overall prevalence of dementia in the entire Medicare program was lower over a 14 year period [15]. However, the experience was different in TM and MA. Prevalence of dementia in TM was consistently lower over time, while prevalence in MA was consistently higher. We also assessed enrollment and clinical characteristics of beneficiaries living with dementia in TM and MA. Compared to TM beneficiaries with dementia, MA beneficiaries were younger; had fewer ADL and IADL limitations and chronic conditions, were more likely to be living in the community during their HRS interview, and less likely to have a proxy respondent. Finally, we found Medicare-only beneficiaries living with dementia were more likely to switch from MA to TM than their counterparts with no cognitive impairment.

The decreasing prevalence over time of dementia in TM and not MA may be explained in part by differences in program enrollment by race. Whites non-Hispanic represent the largest share of Medicare beneficiaries, they are more likely to enroll in TM, and they had a lower prevalence of dementia at the end of the observation period. Consistent with the literature, we found that Blacks non-Hispanic and Hispanics were more likely than Whites non-Hispanic to enroll in MA [17]. Racial/ethnic minorities may be more likely to enroll in MA than Whites non-Hispanic because they are disproportionately low income and MA plans have lower premiums and often extra benefits (e.g., free gym memberships) than TM [18, 19]. Dementia prevalence among Blacks non-Hispanic is twice as high as White non-Hispanic and 1.5 times as high as Hispanics. Thus, during the study period, populations with higher prevalence of dementia were also more likely to enroll in MA.

As MA enrolls a larger share of the Medicare population living with dementia, it is expected that any initial favorable selection would be reduced over time. However, our results suggest that between 2000 and 2014 younger and potentially healthier than average beneficiaries living with dementia enrolled in MA relative to TM, indicating potential favorable selection within MA for the dementia population during this period. This finding is counter to early studies which found that policymakers have succeeded in reducing favorable selection overall [6]. Of importance, prior studies have examined favorable selection for the entire Medicare population. Our analysis focused only on the dementia population, which accounts for ~ 10% of the Medicare population. Studies evaluating favorable selection in MA typically rely on claims and/or encounter data to predict the differences in risk scores and spending of those who switch from TM to MA and those who stay in TM [12, 20, 21]. MA claims data has a limited set of information (e.g., diagnoses) from which to generate risk scores and can be subject to differences in coding intensity among MA plans [22, 23]. In contrast, we used a set of sociodemographic and clinical characteristics, collected via the HRS and linked to both TM and MA beneficiaries. This allowed us to directly compare the health-status of dementia diagnosed populations in TM and MA and examine measures of acuity within the population (i.e., functional activity limitations, and presence of a proxy respondent) that are not available in claims. To test for the robustness of this evidence we stratified by age-group and compared clinical characteristics among people living with dementia in TM and MA. Even among same age-groups and diagnosis (i.e. dementia) there are observable differences, suggesting that MA has enrolled younger and healthier beneficiaries with dementia over this time period. Although our data point to the possibility of favorable selection among beneficiaries with dementia between 2000 and 2014, in this study we do not examine health care expenditures and are unable to definitely answer the question.

The switching analysis in the two-year period between 2012 and 2013 suggests that MA beneficiaries with dementia may experience unmet care needs at higher rates than their counterparts in TM. Although “lock-in” effects may restrict switching from MA to TM and favor switching within MA plans, we observed higher switching among MA beneficiaries living with dementia than among MA beneficiaries with normal cognitive function. Importantly, we did not observe a similar difference between TM beneficiaries living with dementia and TM beneficiaries with normal cognitive function. This evidence could potentially explain some of the differences observed in clinical and sociodemographic characteristics observed between MA and TM beneficiaries living with dementia.

An important contribution of our study is that by using the Langa-Weir approach to predict cognitive function from measures in HRS we are able to use the same method to identify dementia regardless of MA or TM enrollment and address the common problem of undercounting dementia cases in Medicare claims data [15]. Prior studies have estimated the prevalence of dementia in MA but they use a mix of administrative and assessment data [7], which may limit the accuracy of dementia case identification. Instead, we provide nationally representative estimates of dementia prevalence in TM and MA populations. Moreover, several studies have examined the experience of MA beneficiaries with dementia over shorter periods of time. In contrast, our study provides 14 years of data [24]. Finally, in our assessment of disenrollment from MA we can distinguish between disenrollment due to death, or due to switching to TM, addressing some of the limitations of previous studies looking at disenrollment from MA among dementia population [9]. 

Our study has several limitations. First, in 2015 cm transitioned to the ICD-10, which may reduce misclassification of dementia in claims relative to ICD-9 [25]. The accuracy gains in dementia case detection from using the Langa-Weir algorithm may not be as relevant for the period after 2014. Second, we do not distinguish switching between different MA plans due to “lock-in” restrictions in MA, we only consider switching to TM and disenrollment due to death. Third, our findings are descriptive and do not provide any causal evidence of the mechanisms behind the trends in dementia prevalence, MA enrollment and switching, and favorable selection observed in our study. Finally, the relatively small sample size used in the switching analysis (years 2012 and 2013) may restrict the generalizability of the switching results.

## Conclusions

Between 2000 and 2014 the differences in prevalence of dementia between TM and MA vanished as enrollment into MA increased among people living with dementia. There is evidence that MA beneficiaries with dementia have fewer activity of daily living limitations and are overall healthier than their counterparts in TM within the same age-groups. MA beneficiaries with dementia are also more likely to switch to TM compared to beneficiaries without dementia, which may represent the population having unmet care needs.

## Supplementary Information

Below is the link to the electronic supplementary material.

Supplementary Material 1

## Data Availability

The linked datasets analyzed in the current study are not publicly available. Participation in the NIA data LINKAGE Program is required to access data from HRS linked to CMS data.

## Abbreviations

TM: Traditional Medicare
MA: Medicare Advantage
CMS: Centers for Medicare and Medicaid
HRS: Health and Retirement Study
ICD-9: International classification of diseases, ninth revision
ADL: Activities of daily living
IADL: Instrumental activities of daily living
