# Supplementary material for: National trends in the prevalence of dementia in Medicare Advantage and Traditional Medicare
Source: BMC Health Serv Res. 2026 Mar 7;26:519. doi: 10.1186/s12913-025-13404-2 (PMC13081262; doi:10.1186/s12913-025-13404-2)
Supplement: Supplementary file 1 — Supplementary Material 1 [file 12913_2025_13404_MOESM1_ESM.docx]

**Appendix**

**Supplemental** **Table 1:** Characteristics of Medicare Beneficiaries living with Dementia (2000 - 2014, selected in-between years) ^a^

|  | **Traditional Medicare** | | | | | **Medicare Advantage** | | |
| --- | --- | --- | --- | --- | --- | --- | --- | --- |
| **Characteristics** | **2000** | **2006** | **2008** | **2014** | **2000** | **2006** | **2008** | **2014** |
|  | 1,081 | 952 | 780 | 639 | 193 | 190 | 288 | 377 |
| Age, yrs |  |  |  |  |  |  |  |  |
| 65-74 | 243(23.3%) | 228(19.2%) | 184(20.7%) | 98(17.6%) | 47(25.8%) | 50(25.6%) | 70(20.5%) | 64(19.2%) |
| 75-84 | 425(42.0%) | 334(40.6%) | 248(35.0%) | 251(34.1%) | 90(46.2%) | 81(46.1%) | 133(50.5%) | 182(44.9%) |
| >= 85 | 413(34.7%) | 390(40.2) | 348(44.2) | 290(48.3%) | 56(28.0%) | 59(28.2%) | 85(28.9%) | 131(35.9%) |
| Mean(SE) | 81.0(0.3) | 81.8(0.3) | 82.1(0.3) | 83.5(0.4) | 79.8(0.7) | 80.2(0.6) | 80.8(0.4) | 81.6(0.5) |
| Sex |  |  |  |  |  |  |  |  |
| Female | 679(64.0%) | 628(67.6%) | 502(65.6%) | 408(63.4%) | 119(62.2%) | 128(64.2%) | 181(62.7%) | 232(58.2%) |
| Race ^b^ |  |  |  |  |  |  |  |  |
| White non-Hispanic | 655(70.5%) | 584(71.3%) | 491(71.1%) | 409(70.5%) | 128(76.3%) | 105(65.0%) | 153(63.2%) | 202(60.3%) |
| Black non-Hispanic | 281(18.7%) | 250(17.7%) | 172(15.2%) | 130(14.4%) | 41(15.1%) | 46(16.2%) | 92(22.2%) | 97(20.0%) |
| Hispanic | 109(7.4%) | 96(8.5%) | 103(11.7%) | 82(11.7%) | 22(8.2%) | 36(16.4%) | 39(12.9%) | 74(18.2%) |
| Net worth ^c^ |  |  |  |  |  |  |  |  |
| ≤ 32 000 | 559(48.8%) | 460(45.2%) | 377(45.4%) | 273(40.2%) | 71(35.0%) | 88(46.1%) | 112(37.7%) | 176(44.5%) |
| 32 001-120 100 | 257(23.6%) | 212(22.1%) | 138(18.1%) | 129(18.5%) | 53(29.1%) | 39(20.9%) | 62(19.8%) | 63(16.6%) |
| 120 101-300 500 | 148(15.2%) | 127(13.8%) | 108(13.9%) | 104(19.7%) | 50(25.7%) | 27(13.1%) | 55(19.7%) | 75(20.2%) |
| ≥300 501 | 117(12.4%) | 153(18.9%) | 157(22.6%) | 133(21.7%) | 19(10.1%) | 36(19.8%) | 59(22.9%) | 63(18.7%) |
| Mean(SE) | 143,803.0(13,030.9) | 259542.9(40656.9) | 264847.3(26632.4) | 251441.5(24,125.2) | 132,855.8(16,845.0) | 175168.6(23502.7) | 245996.2(44216.2) | 344,060.4(108,561.6) |
| ADL Limitations ^d^ |  |  |  |  |  |  |  |  |
| 0 | 403(35.9%) | 330(35.2%) | 269(35.1%) | 224(37.4%) | 89(43.6%) | 87(43.4%) | 135(47.1%) | 171(46.4%) |
| 1-3 | 336(31.1%) | 288(29.3%) | 225(29.0%) | 179(37.4%) | 59(32.2%) | 54(29.6%) | 77(26.3%) | 105(27.5%) |
| 4-6 | 342(33.0%) | 334(35.4%) | 286(35.9%( | 236(35.9%) | 45(24.2%) | 49(27.0%) | 76(26.7%) | 101(26.0%) |
| Mean(SE) | 2.3(0.1) | 2.4(0.1) | 2.4(0.1) | 2.4(0.1) | 1.9(0.2) | 2.0(0.2) | 1.9(0.1) | 1.9(0.1) |
| IADL Limitations ^e^ |  |  |  |  |  |  |  |  |
| 0 | 275(24.8%) | 223(23.7%) | 191(26.5%) | 152(24.9%) | 65(32.1%) | 62(34.3%) | 88(31.0%) | 122(33.3%) |
| 1-3 | 311(29.8%) | 249(28.3%) | 194(25.3%) | 140(25.5%) | 50(28.0%) | 41(21.5%) | 85(32.2%) | 97(28.6%) |
| 4-5 | 442(45.4%) | 416(48.0%) | 350(48.2%) | 315(49.7%) | 66(39.9%) | 72(44.3%) | 93(36.8%) | 136(38.1%) |
| Mean(SE) | 2.7(0.1) | 2.8(0.1) | 2.8(0.1) | 2.8(0.1) | 2.3(0.2) | 2.5(0.2) | 2.3(0.1) | 2.3(0.1) |
| Cumulative Chronic Health Conditions (ever diagnosed) |  |  |  |  |  |  |  |  |
| 0 | 57(5.7%) | 34(3.9%) | 33(5.1%) | 5(0.7%) | 26(14.0%) | 9(6.1%) | 9(3.2%) | 10(3.4%) |
| 1-3 | 698(65.2%) | 524(58.4%) | 396(54.1%) | 286(49.2%) | 120(61.9%) | 121(67.2%) | 173(64.2%) | 205(56.1%) |
| 4-5 | 174(16.8%) | 201(20.1%) | 153(20.4%) | 157(25.6%) | 20(9.7%) | 21(12.3%) | 44(15.7%) | 80(21.4%) |
| 6-8 | 132(12.3%) | 158(17.6%) | 158(20.3%) | 151(24.5%) | 22(14.4%) | 26(14.5%) | 44(16.9%) | 66(19.2%) |
| Mean(SE) | 2.7(0.1) | 3.1(0.1) | 3.2(0.1) | 3.5(0.1) | 2.5(0.1) | 2.8(0.1) | 3.0(0.1) | 3.2(0.1) |
| Individual Chronic Health Conditions (ever diagnosed) |  |  |  |  |  |  |  |  |
| Stroke | 308(29.8%) | 305(31.4%) | 260(32.6%) | 212(30.9%) | 52(29.8%) | 189(31.6%) | 288(29.8%) | 100(27.5%) |
| Diabetes | 222(19.1%) | 248(24.7%) | 220(26.6%) | 226(33.7%) | 46(23.9%) | 50(23.1%) | 84(28.8%) | 145(38.4%) |
| Heart Disease | 444(41.4%) | 431(45.3%) | 354(44.1%) | 299(46.5%) | 71(39.7%) | 68(38.1%) | 114(40.5%) | 137(37.8%) |
| Hypertension | 643(58.4%) | 678(70.3%) | 574(72.6%) | 511(78.9%) | 100(49.9%) | 135(69.0%) | 200(69.4%) | 305(80.0%) |
| Cancer | 137(13.3%) | 153(17.5%) | 141(18.6%) | 164(25.6%) | 33(16.9%) | 37(17.2%) | 58(21.0%) | 82(20.5%) |
| Lung Disease | 134(13.3%) | 116(12.3%) | 116(14.7%) | 102(17.7%) | 16(9.2%) | 19(12.3%) | 34(11.9%) | 63(16.9%) |
| Psychiatric Problems | 279(26.7%) | 308(33.5%) | 263(34.8%) | 232(36.7%) | 36(20.8%) | 57(32.6%) | 76(29.0%) | 111(29.6%) |
| Arthritis | 754(67.9%) | 695(72.7%) | 590(74.9%) | 515(80.6%) | 111(58.2%) | 128(67.0%) | 204(70.9%) | 280(73.6%) |
| Live in Nursing Home During HRS Interview |  |  |  |  |  |  |  |  |
| yes | 282(29.9%) | 274(30.3%) | 253(31.9%) | 195(26.4%) | 33(20.9%) | 36(20.0%) | 48(17.0%) | 67(14.2%) |
| Respondent Type |  |  |  |  |  |  |  |  |
| Proxy | 557(54.1%) | 428(46.0%) | 373 (47.8% | 310(46.0%) | 77(43.1%) | 81(43.3%) | 104(38.5%) | 135(33.8%) |
| Dual Eligibility Status |  |  |  |  |  |  |  |  |
| Dually Eligible | NA | NA | NA | 408(34.4%) | NA | NA | NA | 130(28.8%) |

Abbreviations: ADL, activities of daily living; IADL, instrumental activities of daily living

Annual Enrollment in Medicare Advantage: months enrolled in TM = 0 and months enrolled in MA >0

Annual Enrollment in Traditional Medicare: months enrolled in TM > 0 and months enrolled in MA =0

a, Percentages in parenthesis are weighted proportions derived using the survey sampling weights of the HRS

b, Due to small number of respondents (< 25) we exclude the count of the category “other” for Race/ethnicity

c Includes all wealth components minus all debt components assessed in HRS.

d, ADL Limitations: eating, transferring, toileting, dressing, bathing, and walking across a room.

e, IADL Limitations: preparing meals, grocery shopping, making phone calls, talking medications, managing money.

**Supplemental** **Table 2:** Clinical characteristics in 2000 and 2014 between TM and MA beneficiaries living with Dementia, stratified by age group

**Panel A:** Age group (65-74 years)

|  | **Traditional Medicare** | | **Medicare Advantage** | |
| --- | --- | --- | --- | --- |
|  | **2000** | **2014** | **2000** | **2014** |
| Net worth^c^ |  |  |  |  |
| ≤ 32,000 | 50.5%(43.5%,57.4%) | 44.3%(32.7%,56.5%) | 41.3%(26.6%,57.7%) | 28.4%(17.9%,41.8%) |
| 32,001-120,100 | 29.9%(23.9%,36.6%) | 23.8%(14.9%,35.8%) | 36.4%(22.6%,53.0%) | 29.6%(18.9%,43.0%) |
| 120,101-300,500 | 11.8%(7.9%,17.2%) | 19.1%(10.8%,31.3%) | 15.7%(7.7%,29.5%) | 26.6%(16.6%,39.8%) |
| ≥300,501 | 7.8%(4.8%,12.5%) | 12.9%(7.0%,22.3%) | 6.6%(1.9%,20.2%) | 15.5%(8.2%,27.3%) |
| Mean(95%CI) | 90,430.7(61,667.3;119,194.2) | 163,790.1(100,268.2;227,312) | 91,409.8(51,932.5,;30,887.1) | 201,554.6(22,361.4;380,747.8) |
| Median(IQR) | 30,000(1,000;91,000) | 51,000(1900;180,500) | 53,000(1,500;114,000) | 25,000(400;13,4020) |
| ADL Limitations ^d^ |  |  |  |  |
| 0 | 49.8%(42.8%,56.7%) | 56.9%(44.7%,68.2%) | 48.9%(33.4%,64.6%) | 48.2%(33.4%,63.2%) |
| 1-3 | 28.5%(22.7%,35.1%) | 19.5%(11.8%,30.6%) | 26.7%(14.9%,43.2%) | 32.7%(19.6%,49.2%) |
| 4-6 | 21.7%(16.3%,28.4%) | 23.6%(15.1%,35.0%) | 24.4%(12.9%,41.1%) | 19.1%(10.3%,32.7%) |
| Mean(95%CI) | 1.6(1.3,1.9) | 1.6(1.1,2.1) | 1.8(1,2.5) | 1.5(1,2.1) |
| IADL Limitations ^e^ |  |  |  |  |
| 0 | 38.4%(31.7%,45.5%) | 40.1%(28.9%,52.5%) | 47.2%(31.3%,63.7%) | 43.7%(29.5%,59.0%) |
| 1-3 | 31.6%(25.3%,38.6%) | 33.6%(22.8%,46.4%) | 20.9%(10.2%,37.8%) | 37.6%(23.9%,53.6%) |
| 4-5 | 30.0%(23.6%,37.3%) | 26.3%(17.5%,37.6%) | 31.9%(18.1%,49.8%) | 18.7%(9.9%,32.6%) |
| Mean(95%CI) | 2(1.7,2.3) | 1.8(1.3,2.2) | 2(1.3,2.7) | 1.4(0.9,1.9) |
| Cumulative Chronic Health Conditions (ever diagnosed) |  |  |  |  |
| 0 | 7.2%(4.4%,11.5%) | 0.5%(0.1%,3.4%) | 10.4%(3.8%,25.2%) | 6.9%(2.1%,20.3%) |
| 1-3 | 66.7%(59.7%,73.1%) | 46.1%(34.0%,58.7%) | 61.2%(44.2%,75.8%) | 49.1%(33.9%,64.4%) |
| 4-5 | 12.2%(8.2%,17.9%) | 26.2%(16.9%,38.3%) | 9.0%(3.0%,24.0%) | 27.1%(15.6%,42.7%) |
| 6-8 | 13.8%(9.5%,19.6%) | 27.2%(17.6%,39.6%) | 19.4%(9.3%,36.1%) | 17.0%(8.8%,30.2%) |
| Mean(95%CI) | 2.6(2.4,2.8) | 3.6(3.3,4) | 3.1(2.5,3.7) | 3.3(2.8,3.7) |
| Live in Nursing Home During HRS Interview |  |  |  |  |
| no | 85.6%(78.7%,90.5%) | 87.3%(78.1%,93.0%) | 86.8%(67.9%,95.4%) | 92.5%(79.8%,97.4%) |
| yes | 14.4%(9.5%,21.3%) | 12.7%(7.0%,21.9%) | 13.2%(4.6%,32.1%) | 7.5%(2.6%,20.2%) |

**Panel B:** Age group (75-84 years)

|  | **Traditional Medicare** | | **Medicare Advantage** | |
| --- | --- | --- | --- | --- |
|  | **2000** | **2014** | **2000** | **2014** |
| Net worth^c^ |  |  |  |  |
| ≤ 32,000 | 48.0%(42.8%,53.2%) | 38.2%(31.7%,45.1%) | 58.6%(44.8%,71.2%) | 44.0%(28.9%,60.3%) |
| 32,001-120,100 | 20.7%(16.8%,25.2%) | 19.8%(15.2%,25.4%) | 12.1%(5.7%,23.9%) | 31.3%(18.8%,47.1%) |
| 120,101-300,500 | 17.3%(13.5%,21.8%) | 19.8%(14.7%,26.2%) | 20.2%(11.1%,34.0%) | 13.4%(6.2%,26.6%) |
| ≥300,501 | 14.1%(10.6%,18.4%) | 22.2%(16.9%,28.5%) | 9.0%(4.0%,19.0%) | 11.3%(4.5%,25.9%) |
| Mean(95%CI) | 152,454.4(119,451.5;185,457.2) | 219,386.2(161,823.7;276,948.6) | 176,176(114,456.3;237,895.6) | 229,821.6(146,288.9;313,354.3) |
| Median(IQR) | 40,000(500;157,000) | 80,000(1,500;244,000) | 94,755(33,200;193,000) | 66,000(2,000;237,002) |
| ADL Limitations ^d^ |  |  |  |  |
| 0 | 36.2%(31.4%,41.2%) | 39.1%(32.6%,46.0%) | 50.2%(38.6%,61.7%) | 52.8%(44.7%,60.7%) |
| 1-3 | 31.7%(27.0%,36.8%) | 32.3%(26.1%,39.1%) | 33.3%(23.0%,45.4%) | 24.2%(18.2%,31.4%) |
| 4-6 | 32.2%(27.3%,37.4%) | 28.6%(22.8%,35.3%) | 16.6%(9.7%,27.0%) | 23.0%(17.0%,30.5%) |
| Mean(95%CI) | 2.3(2.1,2.5) | 2.1(1.8,2.4) | 1.5(1,1.9) | 1.6(1.3,2) |
| IADL Limitations ^e^ |  |  |  |  |
| 0 | 25.6%(21.4%,30.4%) | 26.5%(20.8%,33.0%) | 31.9%(22.1%,43.6%) | 36.9%(29.4%,45.2%) |
| 1-3 | 29.3%(24.7%,34.4%) | 23.4%(18.0%,29.9%) | 35.1%(24.4%,47.4%) | 25.3%(18.5%,33.5%) |
| 4-5 | 45.0%(39.7%,50.5%) | 50.1%(43.0%,57.2%) | 33.1%(22.7%,45.4%) | 37.8%(30.1%,46.2%) |
| Mean(95%CI) | 2.7(2.5,2.9) | 2.8(2.5,3.1) | 2.1(1.6,2.5) | 2.2(1.9,2.6) |
| Cumulative Chronic Health Conditions (ever diagnosed) |  |  |  |  |
| 0 | 6.4%(4.2%,9.6%) | 0.3%(0.0%,2.2%) | 14.3%(7.8%,24.7%) | 2.9%(1.1%,7.5%) |
| 1-3 | 60.1%(54.8%,65.2%) | 39.9%(33.1%,47.0%) | 63.8%(51.8%,74.3%) | 58.5%(50.3%,66.2%) |
| 4-5 | 19.9%(15.9%,24.6%) | 28.4%(22.3%,35.4%) | 6.5%(3.1%,13.0%) | 22.1%(16.3%,29.3%) |
| 6-8 | 13.6%(10.3%,17.8%) | 31.4%(25.1%,38.5%) | 15.4%(8.2%,27.1%) | 16.4%(11.0%,23.8%) |
| Mean(95%CI) | 2.8(2.6,3) | 3.8(3.6,4) | 2.4(2,2.8) | 3.1(2.9,3.4) |
| Live in Nursing Home During HRS Interview |  |  |  |  |
| no | 71.5%(66.1%,76.3%) | 73.8%(66.9%,79.6%) | 83.1%(71.1%,90.7%) | 87.9%(81.5%,92.3%) |
| yes | 28.5%(23.7%,33.9%) | 26.2%(20.4%,33.1%) | 16.9%(9.3%,28.9%) | 12.1%(7.7%,18.5%) |

**Panel C:** Age group ($\geq$ 85 years)

|  | **Traditional Medicare** | | **Medicare Advantage** | |
| --- | --- | --- | --- | --- |
|  | **2000** | **2014** | **2000** | **2014** |
| Net worth^c^ |  |  |  |  |
| ≤ 32,000 | 48.7%(43.6%,53.9%) | 40.1%(34.0%,46.5%) | 35.3%(27.0%,44.7%) | 45.5%(36.3%,54.9%) |
| 32,001-120,100 | 23.0%(18.8%,27.7%) | 15.7%(11.8%,20.5%) | 22.9%(16.1%,31.5%) | 13.7%(8.4%,21.5%) |
| 120,101-300,500 | 14.9%(11.5%,19.1%) | 19.8%(14.3%,26.7%) | 15.2%(9.6%,23.2%) | 19.7%(13.3%,28.4%) |
| ≥300,501 | 13.4%(10.2%,17.4%) | 24.5%(19.5%,30.3%) | 26.6%(19.1%,35.7%) | 21.1%(14.1%,30.4%) |
| Mean(95%CI) | 169,172.2(110,834;227,510.4) | 305,918.3(220,670.8;391,165.8) | 99,517.3(60,584.7;138,449.8) | 563,191.3(0.0;1129,054) |
| Median(IQR) | 38,000(500;142,000) | 75,800(1,500;295,629) | 46,900(500;152,300) | 68,900(1,000;283,000) |
| ADL Limitations ^d^ |  |  |  |  |
| 0 | 26.3%(22.0%,31.2%) | 29.0%(23.5%,35.3%) | 27.8%(17.5%,41.1%) | 37.6%(28.7%,47.3%) |
| 1-3 | 32.1%(27.4%,37.1%) | 25.5%(20.4%,31.3%) | 35.4%(23.0%,50.2%) | 28.9%(21.2%,38.2%) |
| 4-6 | 41.6%(36.6%,46.8%) | 45.5%(39.0%,52.1%) | 36.8%(24.3%,51.2%) | 33.5%(25.5%,42.6%) |
| Mean(95%CI) | 2.8(2.6,3.1) | 2.9(2.6,3.2) | 2.6(2,3.2) | 2.3(1.9,2.8) |
| IADL Limitations ^e^ |  |  |  |  |
| 0 | 14.8%(11.5%,18.8%) | 18.0%(13.5%,23.6%) | 19.0%(10.3%,32.4%) | 22.9%(15.7%,32.1%) |
| 1-3 | 29.1%(24.5%,34.2%) | 23.8%(18.4%,30.2%) | 22.5%(12.9%,36.1%) | 27.6%(19.6%,37.3%) |
| 4-5 | 56.1%(50.8%,61.3%) | 58.2%(51.4%,64.7%) | 58.5%(44.1%,71.7%) | 49.5%(39.8%,59.2%) |
| Mean(95%CI) | 3.2(3,3.4) | 3.2(2.9,3.4) | 3.1(2.5,3.6) | 2.9(2.5,3.3) |
| Cumulative Chronic Health Conditions (ever diagnosed) |  |  |  |  |
| 0 | 3.9%(2.2%,6.6%) | 1.1%(0.3%,3.5%) | 16.8%(8.7%,29.9%) | 1.9%(0.5%,7.5%) |
| 1-3 | 70.4%(65.4%,74.9%) | 56.8%(50.1%,63.3%) | 59.4%(44.9%,72.3%) | 56.8%(47.0%,66.1%) |
| 4-5 | 16.2%(12.8%,20.4%) | 23.4%(18.3%,29.4%) | 15.4%(7.3%,29.6%) | 17.2%(11.2%,25.5%) |
| 6-8 | 9.5%(6.9%,13.0%) | 18.7%(14.1%,24.4%) | 8.4%(3.3%,19.7%) | 24.1%(16.8%,33.3%) |
| Mean(95%CI) | 2.6(2.5,2.8) | 3.3(3.1,3.5) | 2.2(1.8,2.6) | 3.4(3.1,3.7) |
| Live in Nursing Home During HRS Interview |  |  |  |  |
| no | 58.0%(52.8%,63.0%) | 68.6%(62.7%,73.9%) | 65.5%(50.6%,77.8%) | 79.5%(72.4%,85.2%) |
| yes | 42.0%(37.0%,47.2%) | 31.4%(26.1%,37.3%) | 34.5%(22.2%,49.4%) | 20.5%(14.8%,27.6%) |

Abbreviations: ADL, activities of daily living; IADL, instrumental activities of daily living

Annual Enrollment in Medicare Advantage: months enrolled in TM = 0 and months enrolled in MA >0.

Annual Enrollment in Traditional Medicare: months enrolled in TM > 0 and months enrolled in MA =0.

a, Percentages in parenthesis are weighted proportions derived using the survey sampling weights of the HRS.

b, Due to small number of respondents (< 25) we exclude the count of the category “other” for Race/ethnicity.

c Includes all wealth components minus all debt components assessed in HRS.

d, ADL Limitations: eating, transferring, toileting, dressing, bathing, and walking across a room.

e, IADL Limitations: preparing meals, grocery shopping, making phone calls, talking medications, managing money.

**Supplemental** **Table 3:** Dementia Prevalence 2000-2014 (HRS years)

| **Population** |  | **2000** | **2002** | **2004** | **2006** | **2008** | **2010** | **2012** | **2014** |
| --- | --- | --- | --- | --- | --- | --- | --- | --- | --- |
| **Overall Medicare Population** | n | 10,395 | 10,482 | 10,590 | 10,778 | 10,620 | 10,065 | 9,753 | 9,244 |
|  | cases | 1,310 | 1,175 | 1,160 | 1,201 | 1,120 | 1,158 | 1,147 | 1,058 |
|  | (%) | 12% | 11% | 10% | 11% | 10% | 10% | 9% | 9% |
|  | [95% CI] | [.113,.127] | [.101,.113] | [.096,.109] | [.1,.113] | [.0895,.101] | [.0916,.104] | [.088,.1] | [.0835,.0955] |
| **Traditional Medicare Population** | n | 8,037 | 8,585 | 8,701 | 8,296 | 7,519 | 6,935 | 6,424 | 5,765 |
|  | cases | 1,081 | 998 | 975 | 952 | 780 | 826 | 784 | 639 |
|  | (%) | 13% | 11% | 11% | 11% | 9% | 10% | 10% | 9% |
|  | [95% CI] | [.12,.14] | [.1,.12] | [.099,.11] | [.1,.12] | [.085,.099] | [.094,.11] | [.089,.1] | [.078,.093] |
| **Medicare Advantage Population** | n | 2,109 | 1,711 | 1,669 | 2,000 | 2,754 | 2,896 | 3,117 | 3,217 |
|  | cases | 193 | 160 | 156 | 190 | 288 | 295 | 330 | 377 |
|  | (%) | 8% | 9% | 8% | 9% | 10% | 9% | 9% | 10% |
|  | [95% CI] | [.072,.097] | [.074,.1] | [.071,.099] | [.077,.1] | [.087,.11] | [.076,.097] | [.079,.1] | [.085,.11] |

Abbreviations: MA, Medicare Advantage; TM, Traditional Medicare

Annual Enrollment in Medicare Advantage: months enrolled in TM = 0 and months enrolled in MA > 0

Annual Enrollment in Traditional Medicare: months enrolled in TM > 0 and months enrolled in MA = 0

a, Values in squared brackets are weighted proportions derived using the survey sampling weights of the HRS.

b, Due to small number of respondents (< 25) we exclude the count of the category “other” for Race/ethnicity.

**Supplemental** **Table 4:** Plan Switching 2012-2013, by Cognitive Function (Dementia, CIND, Normal) and Dual Eligibility ^a^

|  |  | **2013** | | |  |
| --- | --- | --- | --- | --- | --- |
| **2012** | **Cognitive Function** | **Enrolled in MA 2013 and Alive** | **Enrolled in TM 2013 and Alive** | **Died between 2012-2013** | **Row Total** |
|  | | **No.(row %)** | **No.(row %)** | **No.(row %)** | **No.** |
| **All** | |  |  |  |  |
| **Enrolled in MA 2012 and Alive** | Normal | 2102(94%) | 56(2.5%) | 79(3.5%) | 2237 |
|  | CIND | 669(89.8%) | 29(3.9%) | 47(6.3%) | 745 |
|  | Dementia | 253(74.2%) | 17(5%) | 71(20.8%) | 330 |
| **Enrolled in TM 2012 and Alive** | Normal | 197(4.1%) | 4405(92.2%) | 176(3.7%) | 4778 |
|  | CIND | 63(4.1%) | 1283(92.2%) | 138(3.7%) | 1484 |
|  | Dementia | 27(3.3%) | 607(73.2%) | 195(23.5%) | 829 |
| **Non-dual eligible population** | |  |  |  |  |
| **Enrolled in MA 2012 and Alive** | Normal | 1928(94.3%) | 49(2.4%) | 68(3.3%) | 2045 |
|  | CIND | 539(89.4%) | 23(3.8%) | 41(6.8%) | 603 |
|  | Dementia | 181(72.4%) | 14(5.6%) | 55(22%) | 250 |
| **Enrolled in TM 2012 and Alive** | Normal | 166(3.8%) | 4088(92.6%) | 159(3.6%) | 4413 |
|  | CIND | 44(3.8%) | 1009(86.7%) | 111(9.5%) | 1164 |
|  | Dementia | 14(2.6%) | 387(72.6%) | 132(24.8%) | 533 |
| **Dual eligible population** | |  |  |  |  |
| **Enrolled in MA 2012 and Alive** | Normal | 174(90.6%) | 7(3.6%) | 11(5.7%) | 192 |
|  | CIND | 130(91.5%) | 6(4.2%) | 6(4.2%) | 142 |
|  | AD/ADRD | 72(79.1%) | 3(3.3%) | 16(17.6%) | 91 |
| **Enrolled in TM 2012 and Alive** | Normal | 31(8.5%) | 317(86.8%) | 17(4.7%) | 365 |
|  | CIND | 19(5.9%) | 274(85.6%) | 27(8.4%) | 320 |
|  | AD/ADRD | 13(4.4%) | 220(74.3%) | 63(21.3%) | 296 |

Abbreviations: MA, Medicare Advantage; TM, Traditional Medicare; CIND, cognitive impairment not dementia, AD/ADRD, Alzheimer’s disease and Alzheimer’s related Dementia

^a^ Dually eligible: Medicare beneficiaries identified to be eligible to receive Medicaid benefits for at least one month during 2012.

**Supplemental** **Figure 1:** MA Enrollment between 2000-2014, by Cognitive Function and Race ^a, b^

Cognitive Function Categories (Dementia, CIND, Normal)


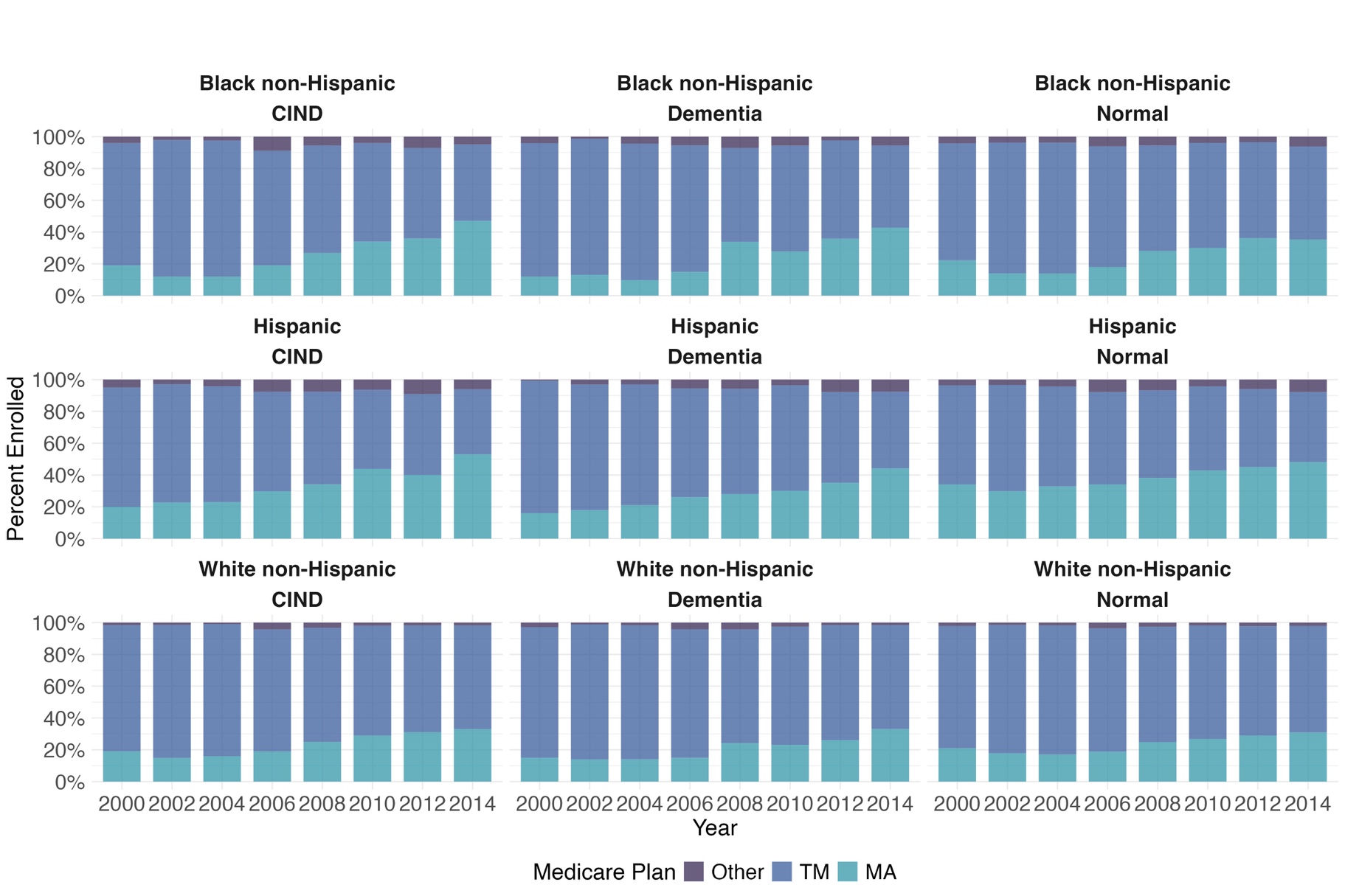


Abbreviations: MA, Medicare Advantage; TM, Traditional Medicare

Annual Enrollment in Medicare Advantage: months enrolled in TM = 0 and months enrolled in MA >0

Annual Enrollment in Traditional Medicare: months enrolled in TM > 0 and months enrolled in MA =0

“Other” refers to cases that were not classified as TM or MA based on the criteria for enrollment.

a, Percentages are weighted proportions derived using the survey sampling weights of the HRS.

b, Due to small number of respondents (< 25) we exclude the count of the category “other” for Race/ethnicity.

**Supplemental** **Figure 2:** MA Enrollment between 2000-2014, by Cognitive Function and Race ^a, b^

Sensitivity to Enrollment Criteria (12 months in a year); Cognitive Function Categories (AD/ADRD, Normal)


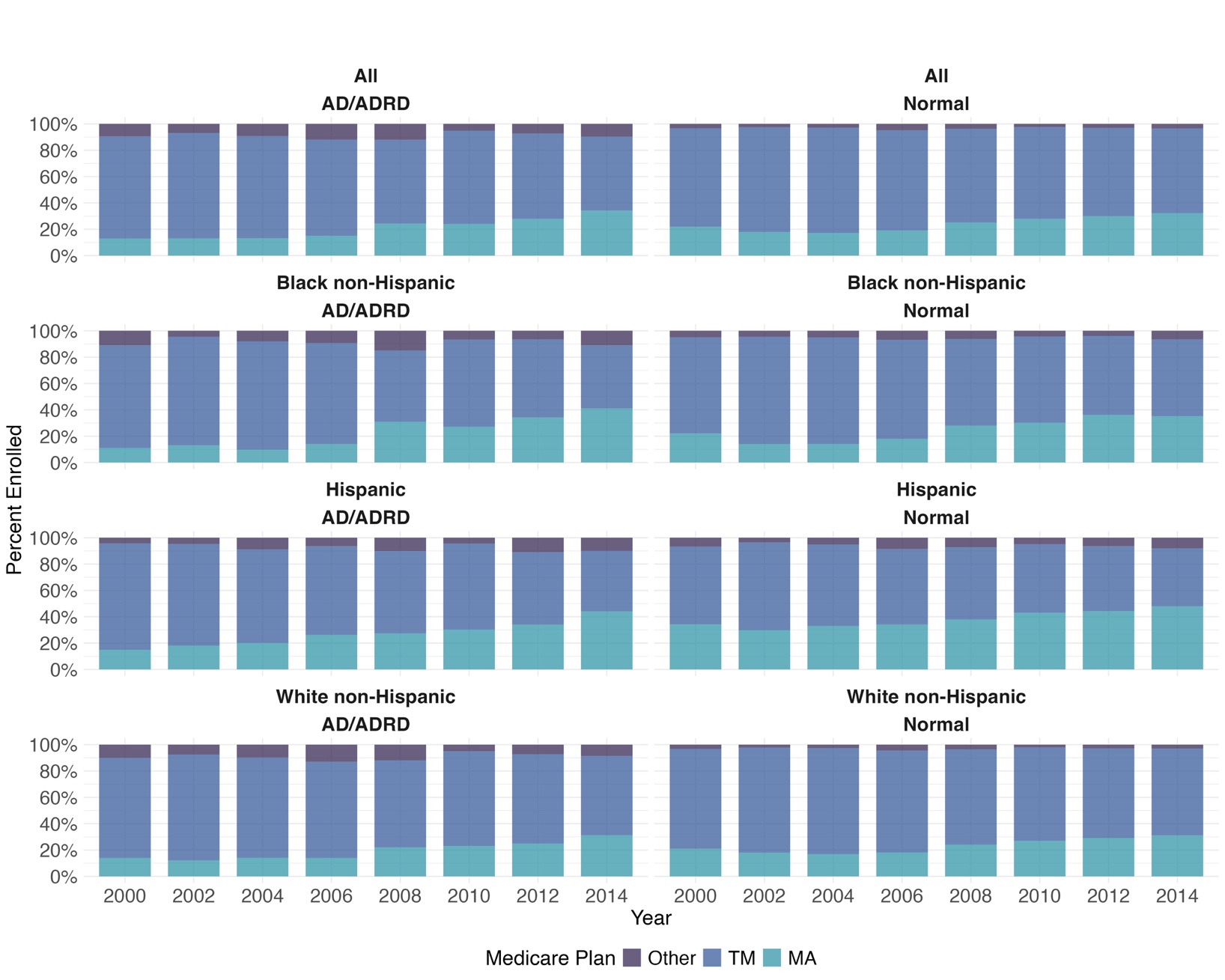


Abbreviations: MA, Medicare Advantage; TM, Traditional Medicare

Annual Enrollment in Medicare Advantage: months enrolled in TM = 0 and months enrolled in MA =12

Annual Enrollment in Traditional Medicare: months enrolled in TM = 12 and months enrolled in MA =0

“Other” refers to cases that were not classified as TM or MA based on the criteria for enrollment.

a, Percentages are weighted proportions derived using the survey sampling weights of the HRS.

b, Due to small number of respondents (< 25) we exclude the count of the category “other” for Race/ethnicity.
